# Are non-constant rates and non-proportional treatment effects accounted for in the design and analysis of randomised controlled trials? A review of current practice

**DOI:** 10.1186/s12874-019-0749-1

**Published:** 2019-05-16

**Authors:** Kim Jachno, Stephane Heritier, Rory Wolfe

**Affiliations:** School of Public Health and Preventive Medicine, Monash University, Level 4, 553 St. Kilda Road, Melbourne, 3004 Australia

**Keywords:** Randomised controlled trial, Time-to-event outcome, Proportional hazards, Event rates, Trial reporting, Sample size calculation

## Abstract

**Background:**

Most clinical trials with time-to-event primary outcomes are designed assuming constant event rates and proportional hazards over time. Non-constant event rates and non-proportional hazards are seen increasingly frequently in trials. The objectives of this review were firstly to identify whether non-constant event rates and time-dependent treatment effects were allowed for in sample size calculations of trials, and secondly to assess the methods used for the analysis and reporting of time-to-event outcomes including how researchers accounted for non-proportional treatment effects.

**Methods:**

We reviewed all original reports published between January and June 2017 in four high impact medical journals for trials for which the primary outcome involved time-to-event analysis. We recorded the methods used to analyse and present the main outcomes of the trial and assessed the reporting of assumptions underlying these methods. The sample size calculation was reviewed to see if the effect of either non-constant hazard rates or anticipated non-proportionality of the treatment effect was allowed for during the trial design.

**Results:**

From 446 original reports we identified 66 trials with a time-to-event primary outcome encompassing trial start dates from July 1995 to November 2014. The majority of these trials (73%) had sample size calculations that used standard formulae with a minority of trials (11%) using simulation for anticipated changing event rates and/or non-proportional hazards. Well-established analytical methods, Kaplan-Meier curves (98%), the log rank test (88%) and the Cox proportional hazards model (97%), were used almost exclusively for the main outcome. Parametric regression models were considered in 11% of the reports. Of the trials reporting inference from the Cox model, only 11% reported any results of testing the assumption of proportional hazards.

**Conclusions:**

Our review confirmed that when designing trials with time-to-event primary outcomes, methodologies assuming constant event rates and proportional hazards were predominantly used despite potential efficiencies in sample size needed or power achieved using alternative methods. The Cox proportional hazards model was used almost exclusively to present inferential results, yet testing and reporting of the pivotal assumption underpinning this estimation method was lacking.

**Electronic supplementary material:**

The online version of this article (10.1186/s12874-019-0749-1) contains supplementary material, which is available to authorized users.

## Background

Time-to-event analysis, or survival analysis, has become the most widely utilized analytical method in research articles in leading general medical journals over the past two decades [1]. These analytical methods compare the duration of time until an event of interest occurs between different intervention groups. Randomised controlled trials (RCTs) provide the highest level of evidence on which to base decisions regarding the use of health interventions in humans. The Cox proportional hazards (PH) model [2] has become ubiquitous as the primary method for assessing treatment effects in RCTs with time-to-event outcomes. Its usage is matched only by the log rank test and Kaplan-Meier curves. Despite the popularity of the Cox PH model to estimate treatment effects, consideration of the fundamental assumption of proportional hazards is not always considered and reported [3].

Over the past two decades, the work and publications of the Consolidated Standards of Reporting Trials (CONSORT) group have encouraged the adoption of guidelines to report RCTs and other research designs [4–6]. Concurrently, there has been a range of policies issued by funding bodies and medical research publishers to enhance the quality, accountability and transparency of clinical trial design and reporting [7, 8]. In September 2004, the International Committee of Medical Journal Editors (ICMJE) disseminated a policy that pre-registration in a public trials registry would be required as a condition of consideration for publication for any trial starting from July 2005 [8]. Partly as a result of these improvements in regulatory oversight, trials are generally larger, and treatment effects are being evaluated for longer [9, 10] and as a consequence non-proportional hazards are detected more frequently [11]. Additionally, trials investigating different therapy modalities, such as immunotherapy compared to chemotherapy, or surgical compared to nonsurgical approaches [12], and the increased use of composite endpoints could also be reasons to anticipate treatment effects that vary over time. The summary hazard ratio (HR) effect measure from the Cox PH model may be less than ideal for decision making when treatment effects change over time [13]. By assuming the effect of treatment is always in the same direction, the HR from the Cox model has the potential to over or underestimate the magnitude of the treatment effect at any given time. Of more concern, if the effect of treatment changes direction over time then the true efficacy of a treatment, or safety issues with the treatment may be missed entirely if a summary HR is relied on.

When designing trials with time-to-event outcomes, sample size formulae exist to inform the required number of events needed to compare two survival distributions with a target effect size and desired power. The number of participants needed to be recruited is then calculated using expected event rates (the hazard), length of recruitment and follow up stages, any loss to follow up, administrative censoring and other logistical considerations in order to observe the number of events required. The most widely used sample size calculation methods to determine the number of events needed are based on the non-parametric log rank test [14, 15] which is most powerful for detecting alternative hypotheses when the hazards are proportional but makes no assumption about the distribution of the baseline hazard function. Alternative methods are based on the difference between two exponential survival functions [16, 17] which assumes proportional hazards as well as the more restrictive assumption of a constant baseline hazard function. Almost equivalently, the sample size formula derived for the HR from a Cox model [18] assumes proportional hazards between the different arms of the trial, but does not make any assumptions about the shape of baseline hazard function. While the Cox model does not assume a constant baseline hazard function, the sample size calculations based upon it yield almost equivalent number of events required to calculations assuming exponential survival rates. However, the shape of the hazard will influence the times at which those events are observed, and hence this needs to be considered together with other logistical considerations such as censoring rates in order to ascertain how many participants need to be recruited to the trial.

In the past two decades, several sample size methods have been proposed that acknowledge that the assumptions of proportional hazards and constant event rates may be too restrictive. These have included incorporating Fleming-Harrington weights [19, 20], allowing for non-proportionality to be specified as a series of piecewise exponential ‘stages’ within a trial [21], or sample size calculations that address specific types of non-proportionality such as lag to effect [20]. Parametric modelling approaches that allow for non-constant event rates such as the Weibull distribution [22, 23] or the generalized gamma distribution [24] have also been proposed. Simulation strategies can be used to empirically determine the sample size required and this approach enables either or both of (i) event rates assumed to change over time and (ii) anticipated non-proportionality of the treatment effect [25]. However, simulation requires a higher degree of programming skill and prior specification of more parameters in order to arrive at a final sample size. The uptake in trial practice of these alternative theoretical or empirical methods of sample size calculation has not been assessed to date.

There are three main approaches to analyzing time-to-event data involving non-parametric, semi-parametric and parametric models. Non-parametric methods such as the Kaplan-Meier method [26], or the method of Nelson [27] and Aalen [28] account for censoring and other characteristics of time-to-event data without making assumptions about the distribution of the event times through the hazard function or how the covariates affect event occurrence. The semi-parametric Cox model makes no assumption about the shape of the hazard function but covariates are assumed to have a multiplicative effect on the hazard. Parametric modelling alternatives to the Cox model such as the exponential-, Weibull- and Gompertz-distributed models assume a specific form for the hazard function as well as making the PH assumption. Other parametric models such as accelerated failure time models utilizing the Weibull and log-logistic distributions, or more recently developed fully flexible spline-based approaches [29, 30] are alternatives to semi-parametric modelling which may enable more clinically useful measures of absolute, as well as relative risk and measures of treatment effect that can be presented as either risk-based (hazard) or time based measures such as the absolute difference in mean survival time due to treatment. Models with a fully specified hazard function also enable easier accounting for, and presentation of time-dependent effects [31].

Previous reviews of survival analysis methodology have found that awareness and reporting of the proportional hazards assumption when using the Cox model has been lacking [32, 33]. Current methods for assessing the validity of the PH assumption include visual assessments and analytical tests. Graphical methods to assess proportionality involve inspection of log-transformed cumulative hazard functions [34] or scaled Schoenfeld residuals [35] against log-transformed time to observe equal slopes or horizontal lines when the PH assumption holds. Scaled Schoenfeld residuals can also be used in an analytical test for trend of non-zero slope against time - the Grambsch and Therneau test [36]. Another analytical method for assessing departures from proportionality is to create an interaction of treatment and time and inspect the significance of that time-dependent covariate [2] when included in a Cox model. However, all of these methods for assessing non-proportionality have some limitations, lacking power to detect some non-linear trends, or involving subjectivity or a particular form of departure from the PH assumption in the process [37].

The aims of this review were to assess the methods currently utilized to (i) accommodate anticipated non-constant treatment effects or event rates during the design phase, and (ii) account for non-proportional treatment effects over time during the analysis phase of trials involving time-to-event outcomes. When Cox models were used, we aimed to document whether there was evidence of an awareness of the underlying PH assumption, along with the any planned or reported PH testing, in either the main trial report or supplementary documentation. With the increased emphasis on improving the adequacy of reporting of results from trials over the past two decades, we also examined whether guidelines or policies may have had an impact on trial conduct.

## Methods

**Fig. 1:**
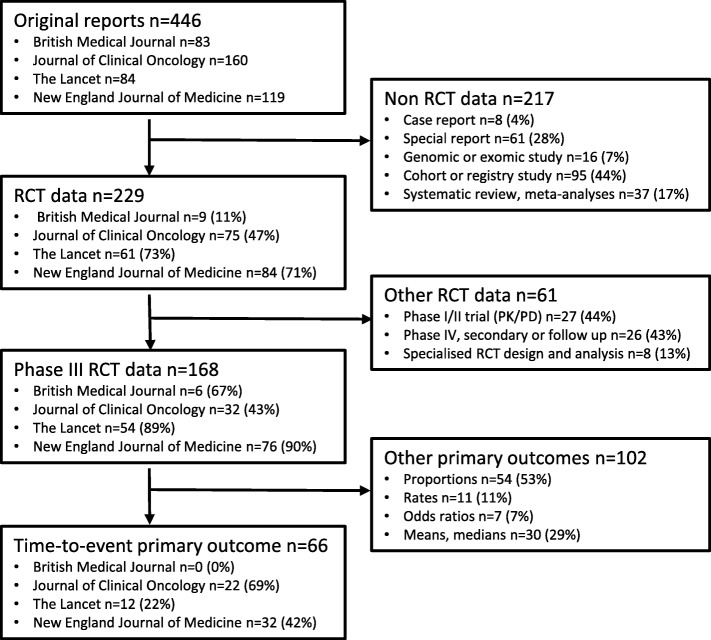
Study design and primary focus of original reports included in this review. The boxes on the left side contain a listing of the classification of the 446 original reports divided into the numbers (n) from each of the four journals reviewed. Percentages in the subsequent boxes use the journal-specific number (n) from the previous box as the reference. The boxes on the right side are the different exclusion criteria applied to the original reports to obtain the final cohort of 66 Phase III RCTs with time to event primary outcomes reviewed. Percentages in each exclusion criteria box use the total number (n) of exclusions at that step as the reference

All original reports published between January and June 2017 in three high impact general medical journals, the *New England Journal of Medicine*, the *British Medical Journal* and *The Lancet*, and one high impact specialized oncology journal, the *Journal of Clinical Oncology*, were considered. Initial screening excluded reports that were not based on data obtained from RCTs such as case reports and cohort studies, genomic and exomic analyses, systematic reviews, special reports or meta-analyses. Secondary screening then excluded articles that were reports early in the pipeline of drug development primarily investigating safety, pharmacokinetics and pharmacodynamics (Phase I and II trials), and reports of RCT data that were follow up or secondary reports (Phase IV trials). Finally, Phase III RCTs where the primary outcome was not a time-to-event endpoint, and reports requiring specialized trial design and analysis methodologies such as cluster randomised trials, or those involving crossover designs were excluded (see Fig. 1).

For each included trial we (KJ) recorded methodological approaches to calculating the sample size, and the clarity and completeness of the reporting of the assumptions that underpinned the sample size calculation. We noted time-to-event methods used for analysis and presentation. For trials using the Cox PH model, we recorded whether the PH assumption was acknowledged and investigated, the test(s) used and whether results of these investigations were detailed anywhere in the main report, attached protocols or other supplementary information. Trial registration information was collected for all trials and the information from the appropriate registry was used in addition to dates provided in the report to determine nominated trial start and end dates for the primary outcome. The publication date used was the issue publication date.

## Results

There were 446 original reports published in the four selected journals during the review period and 66 of these reports were trials with a primary time-to-event outcome (Fig. 1). A citation listing of the final 66 trials is provided as additional material (see Additional file 1). The dataset of the final categories determined for the statistical approaches used in the trials is also provided (see Additional file 2).

### Description and summary findings of the statistical approaches used in trials

The statistical method characteristics of the trials in this review are summarized in Table 1. For the design phase of the trials, sample size approaches based on formulae involving a time-to-event outcome were categorized as either the log rank test, exponential survival distributions, the Cox PH model or simulation categories. Sample size approaches based on formulae involving a binary outcome at a pre-specified time point such as detecting a difference in proportions of event occurrence between the different arms of the trial were categorized as difference in proportion.

**Table 1:** Reported characteristics of the trials

Reported trial characteristic			*N* (%)
Sample size calculation approach			
	Log rank test		40 (61%)
	Cox model beta coefficient		4 (6%)
	Exponentially distributed survival		4 (6%)
	Simulation		7 (11%)
	Difference in proportions		6 (9%)
	Unclear		5 (6%)
Time-to-event analytical methods^a^			
	Non-parametric log rank test		58 (88%)
	Cox PH model		64 (97%)
	Parametric regression		7 (11%)
	Landmark analysis		7 (11%)
Proportional hazards (PH) assumption^b^			
	PH assumption acknowledged		34 (53%)
	PH testing methods documented		31 (48%)
		Analytical test methods	10 (16%)
		Visual assessment methods	6 (9%)
		Visual and analytical methods	7 (11%)
		Unspecified	8 (13%)

^a^Trials typically presented more than one analytical method

^b^for the 64 studies where Cox PH model used

For the analysis phase of the trial, the time-to-event methods that were identified included the use of the non-parametric log rank test, the semiparametric Cox PH model, parametric regression models and landmark analysis approaches for providing multiple estimates of treatment effect. For trials where the Cox PH model was used, there was a further assessment of any acknowledgement of the underlying proportional hazards assumption, and details, if provided, about the method(s) planned to test the assumption.

**Fig. 2:**
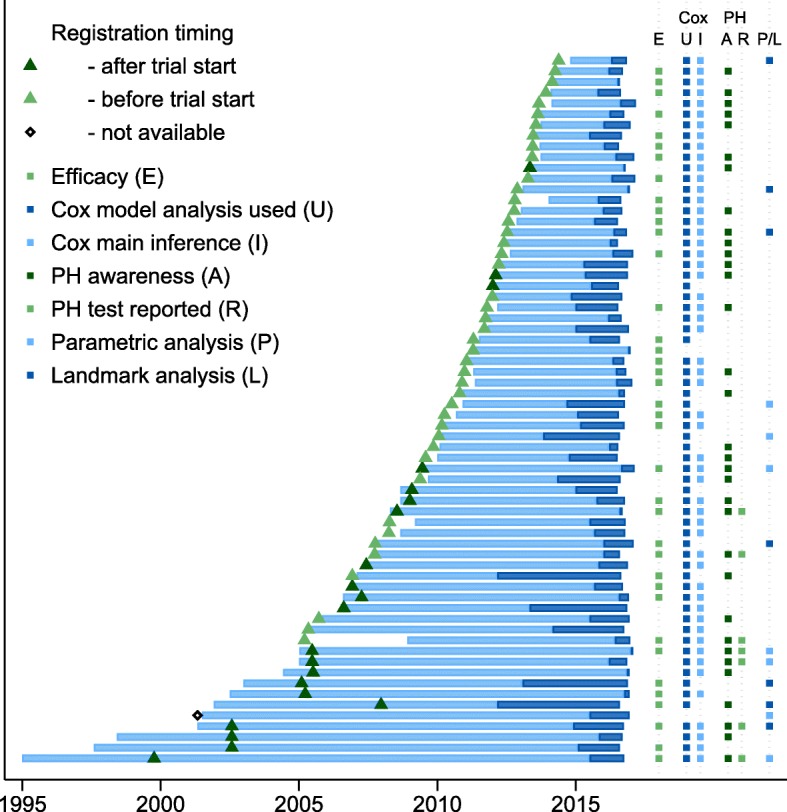
Summary presentation of the findings of the review. Trial duration (years), between nominated start date and completion date, is indicated by the lighter shaded horizontal bars. Duration of time between completion and publication data is indicated by the darker shaded horizontal bars. Time of trial registration is shown by the triangles with lighter and darker shading indicating registration before and after nominated trial start date. Columns on the right side represent the determinations of trial characteristics for this review, including a trial reporting efficacy (E) of the primary outcome, the Cox PH model usage (U) in the report and presentation of the hazard ratio as the main inferential (I) finding. For trials using Cox analysis, the determinations of the awareness (A) and reporting (R) of the proportional hazards assumption for each trial is presented. Planned or presented usage of alternative regression models to the Cox PH model such as parametric or landmark (P/L) analysis is shown in the final column

Figure 2 presents a summary of our findings. Trial duration and time between trial completion and publication are represented by the lighter and darker horizontal bars respectively. The trials had start dates or registration dates in public databases stretching over a period of nearly two decades from July 1995 through November 2014, providing a means to assess if there have been any changes in trial design and reporting over that period. Trial registration timing relative to the start of recruitment is indicated by the triangles. Following the policy adopted by most major medical journal requiring trials to be prospectively registered, changes in timeliness of the trial registration process is evident. No trials which began prior to July 2005 had been registered prior to the nominated start date of the trial, with the clear majority of trials after July 2005 being registered prior to, or in a timely manner after, the nominated start date of the trial.

There was no discernible pattern of change of trials reporting efficacy of primary outcome over time with the 38 (58%) RCTs reporting significant primary outcome findings being evenly spread throughout the two decades’ starting time encapsulated within this review (Fig. 2, column E).

### Designing trials - sample size calculations

There were 7/66 (11%) calculations based on simulation for predicted non-constant event rates over the course of the trial or to allow for an anticipated cure proportion or other non-proportional treatment effect in the trial. Methods that explicitly assume PH, or are maximally powerful under a PH assumption, were used in the majority (*n* = 48/66; 73%) of the sample size calculations. Among these, calculation based on the log rank test was most common (*n* = 40/48; 83%) noting that this utilizes ordered event times and is derived assuming a constant treatment effect over time. Other calculations were based on methods assuming PH for the treatment effect - either through assuming a difference between exponential survival distributions (*n* = 4/48; 8%) with the additional assumption of constant hazard functions, or the beta coefficient (HR) of a Cox model (*n* = 4/48; 8%) which does not make any assumptions about the shape of the baseline hazard function.

There were six trials which used a sample size calculation based on analysis of a difference in proportions of event occurrence in the different arms of the trial at a pre-specified fixed time. For three of these trials, this was justified by specified dual aims for the primary endpoint, (i) to show non-inferiority at a pre-specified time point using a difference in proportions, and (ii) to show superiority of the experimental treatment of interest using time-to-event methods. There were five reports where the basis for the sample size calculation was unclear.

## Methods for the presentation and inference of results

For the graphical presentation of the primary outcome results, in 65/66 trials (98%) there was either a Kaplan-Meier survival plot or its reciprocal, a cumulative incidence plot. The Cox PH model was reported in 64/66 trials (97%) and the non-parametric log rank test was reported in 58/66 trials (88%; see Table 1). The dominance of the Cox PH model as a means to assess time-to-event outcomes, and in particular as the main inferential finding of the reports in this review is evident in Fig. 2 (columns U and I).

There were seven trials that planned to use parametric regression-based modelling approaches that could account for treatment effects changing over time (Table 1 and Fig. 2, column P/L). Six trials used parametric methods as well as the Cox PH method and one trial used parametric regression as the only inferential method. Regression approaches used were Weibull and flexible spline-based regression models that accounted explicitly for event rates being dependent on time, and exponential regression models using a dichotomous change point to allow for the effect of treatment to differ in two pre-specified stages. Seven trials out of 66 (11%) used the Cox model and also performed secondary ‘landmark’ analyses of the primary outcome presenting multiple estimates of the treatment effect for subsets of patients contingent on reaching intermediate event indicators, such as survival to one year or complete response in a biomarker assay.

### Awareness of the PH assumption

About half of the reports (34/64; 53%) using the Cox model indicated an awareness of the importance of the PH assumption (Table 1 and Fig. 2, column A), and a similar proportion (31/64; 48%) included details of planned testing to check for any departures from proportionality in either the main report, attached supplementary information or any additional published protocols or statistical analysis plans referenced by the report. Analytical tests (17/64; 27%), either a time by treatment interaction in the Cox model or the Grambsch-Therneau test, were the most planned method of assessing for potential changing treatment effects over time, followed by visual means (13/64; 20%). Only seven reports (11%) explicitly presented the results of either visual or analytical tests of the assumption (Fig. 2, column R).

### Influences on reporting assessment of the PH assumption

Comprehensive reporting of the PH assumption was more likely to occur when statistically significant results were being presented. Six of the seven trials reporting results of the PH testing also reported a statistically significant effect of treatment on the primary outcome. Of the 27 trials where there was an awareness but not reporting of the PH assumption, 22 trials (81%) used the Cox model as the main inferential finding with half of these presenting significant findings (Fig. 2, column I). In the 30 trials where there was no mention of the PH assumption, 24 trials (80%) presented the Cox model as the main inferential result, with 14 of these significant findings and 10 non-significant findings.

We expected that guidelines such as the CONSORT statement and improved regulatory oversight would have led to an increased consideration to plan and report investigations of the PH assumption over time. Unexpectedly, reporting of PH assumption test results was only seen in trials that commenced prior to June 2009. This might be explained by trials of longer planned duration having a greater awareness of the potential for time-dependent treatment effects to manifest, and hence be more likely to explicitly report results of tests of the PH assumption. However, it is of concern that there was no evidence of increased awareness and reporting of investigation of the PH assumption in trials initiated more recently, irrespective of the planned duration of the trial.

## Discussion

This review assessed design and analysis of RCTs with time-to-event primary outcomes in an era in which non-constant event rates and non-proportional treatment effects are encountered more frequently. Our findings are now discussed alongside previous reviews of reporting of RCTs involving time-to-event primary outcomes and other relevant literature.

### Sample size calculations – adequacy of reporting

Previous reviews have assessed the sample size calculations for a mix of continuous and binary as well as time-to-event outcomes [38, 39]. These reviews concluded that whilst reporting of sample size calculations has improved over time as a result of more stringent requirements imposed by journals and the provision of guidelines such as the CONSORT statement, there were still inadequacies in the assumptions reported and that post hoc modification of sample size parameters was frequent. In our review we too found that initial sample size calculations could have been more adequately reported: the number of participants in the trial was often adjusted for appropriate reasons such as interim analysis, important secondary analysis, or loss to follow up without clear demarcation between the number of events required using the sample size formula and the number of participants to be recruited. We found encouraging signs that researchers are beginning to anticipate the impacts of non-proportional hazards and changing event rates on sample size calculations evidenced by seven trials using simulation-based procedures for their determination of sample size. No trials in our review used more recently proposed modified sample size calculations to allow for anticipated cure proportions [40] or lag times until full treatment effect [20, 41] as could be anticipated in many of the immunotherapy-based treatments under assessment in oncology trials.

### Modelling approaches – changes in recent years

Our review highlights a gradual change over recent decades in the modelling approaches used by general medical and oncology researchers to assess treatment effects on time-to-event outcomes. A review of survival analyses in four cancer journals published during 1991 [32], reported that the log rank test was used to assess treatment differences in 84/113 (74%) whereas only 4/113 (4%) trials used the Cox PH model. No parametric models were used to assess the treatment effect in that review. Over a decade later, another review of 274 trials in major cancer journals published during 2004 [33] found that the log rank test was used in 63% of studies with the Cox model being used in 51% of studies to report the treatment effect. Again, no parametric models were used. Similarly, a review of reports published in five oncology journals during 2015 found that the log rank test was used in 66% of studies with the Cox model being used in 88% of studies to report the treatment effect, and there was no reported use of parametric modelling approaches [42]. In our review, the log rank test was used in 88% of studies, the Cox model in 97% of studies, and parametric modelling approaches were proposed or used in 11% of trials. We also noted that additional landmark analysis was used in 11% of the trials, indicating recognition by the authors that one summary measure of treatment effect did not fully describe the trial findings.

### Assessing for treatment effects that are over time-dependent

Despite the widespread use of the Cox proportional hazards model in medical research, awareness and testing for non-proportionality has not yet become systematic. In the 1995 review of four cancer journals, only 2 (5%) of 43 papers which used the Cox model mentioned that the PH assumption was verified whilst in 2004, one of 64 (2%) usages of a Cox model reported verifying the PH assumption [32, 33]. More recently, a review of trials from five journals published during 2014 [3] found that there was evidence of non-proportionality in 13/54 trials (24%) determined by digitally recreating the individual patient data from the published Kaplan–Meier curves; however, there was no indication of the number of trials in which the PH assumption was assessed in the original reports for that review. A review of survival analysis reporting in the same or similar journals [42] published in 2015 found that only 2/32 (7%) trials using the Cox PH model reported testing for the PH assumption. Our review found the highest reporting rate of 7/64 (11%) which suggests that guidelines to improve the reporting of results may be having an effect but there is still considerable room for improvement.

### Success of guidelines and policies for improving the quality of reporting

The success of journal guidelines and requirements for improving the quality of the reporting of trials is evident in the change in timeliness of trial registrations in our review. The four reviewed journals are either members of the ICMJE or adopted the July 2004 policy requiring pre-trial public registration as a condition of publication for trials commencing from July 2005 with trials beginning prior to that date able to register under an exemption clause by September 2005. No trials which began prior to July 2005 had been registered prior to the nominated start date of the trial, whereas the clear majority of trials after July 2005 had been registered prior to, or shortly after the nominated start date of the trial (Fig. 2). This success stands in contrast to the assessment and reporting of the PH assumption in Cox models, resulting in renewed calls made by others [43], and echoed here by us, for the reviewers, journal editors, regulators and funders of research to demand enhanced content in reports and associated supplementary documentation in order to improve trial reproducibility and interpretation.

## Conclusions

In this review, we explored whether researchers account for non-constant event rates and non-proportional treatment effects during the design, analysis and reporting phases of randomised trials. The insights we derive are timely as health research has entered an era in which trials are being conducted for longer durations and are often adequately powered to evaluate the durability of treatment effects over time. Longer trials make the PH assumption increasingly unrealistic over the entire study duration. In addition, treatment effects that change over time are more likely to be encountered in trials due to the increased use of composite endpoints, and due to the nature of interventions that are now employed in late stage oncology trials. The journals included in this review were all high impact journals that have emphasized the CONSORT guidelines as part of their submission requirements yet the quality of the reporting over the past two decades has been consistently less than optimal. These major medical journals have rigorous statistical review policies and require protocols and other supplementary documents to accompany their original reports of RCTs. This enhanced comprehensiveness of reporting gives investigators adequate scope for completeness and precision in the reporting of trial results.

## Additional files

Additional file 1: Listing of the sixty-six randomised clinical trials in this review. A citation listing by journal. (DOCX 27 kb)

Additional file 2: Determination of the characteristics of the sixty-six randomised clinical trials in this review. Dataset containing the final determinations of trial characteristics. (XLS 63 kb)

## Abbreviations

CONSORT: Consolidated Standards of Reporting Trials
HR: Hazard ratio
ICMJE: International Committee of Medical Journal Editors
PH: Proportional hazards
RCT: Randomised controlled trial
