# Supplementary material for: Are non-constant rates and non-proportional treatment effects accounted for in the design and analysis of randomised controlled trials? A review of current practice
Source: BMC Med Res Methodol. 2019 May 16;19:103. doi: 10.1186/s12874-019-0749-1 (PMC6524252; doi:10.1186/s12874-019-0749-1)
Supplement: Supplementary file 1 — Listing of the sixty-six randomised clinical trials in this review. A citation listing by journal. (DOCX 27 kb) [file 12874_2019_749_MOESM1_ESM.docx]

SUPPLEMENTARY MATERIAL

ARE NON-CONSTANT AND NON-PROPORTIONAL TREATMENT EFFECTS ACCOUNTED FOR IN THE DESIGN AND ANALYSIS OF RANDOMISED CONTROLLED TRIALS? A REVIEW OF CURRENT PRACTICE

Supplementary Table 1: Citation references for the 66 articles included in the review:

| **Journal of Clinical Oncology** |
| --- |
| Powles T, Huddart RA, Elliott T, Sarker SJ, Ackerman C, Jones R, Hussain S, Crabb S, Jagdev S, Chester J, Hilman S, Beresford M, Macdonald G, Santhanam S, Frew JA, Stockdale A, Hughes S, Berney D, Chowdhury S. Phase III, Double-Blind, Randomized Trial That Compared Maintenance Lapatinib Versus Placebo After First-Line Chemotherapy in Patients With Human Epidermal Growth Factor Receptor 1/2-Positive Metastatic Bladder Cancer. Pubmed ID 28034079. J Clin Oncol. 2017 Jan;35(1):48-55. doi: 10.1200/JCO.2015.66.3468. |
| Beer TM, Kwon ED, Drake CG, Fizazi K, Logothetis C, Gravis G, Ganju V, Polikoff J, Saad F, Humanski P, Piulats JM, Gonzalez Mella P, Ng SS, Jaeger D, Parnis FX, Franke FA, Puente J, Carvajal R, Sengeløv L, McHenry MB, Varma A, van den Eertwegh AJ, et al. Randomized, Double-Blind, Phase III Trial of Ipilimumab Versus Placebo in Asymptomatic or Minimally Symptomatic Patients With Metastatic Chemotherapy-Naive Castration-Resistant Prostate Cancer. Pubmed ID 28034081. J Clin Oncol. 2017 Jan;35(1):40-47. doi: 10.1200/JCO.2016.69.1584. |
| Perez EA, Barrios C, Eiermann W, Toi M, Im YH, Conte P, Martin M, Pienkowski T, Pivot X, Burris H 3rd, Petersen JA, Stanzel S, Strasak A, Patre M, Ellis P. Trastuzumab Emtansine With or Without Pertuzumab Versus Trastuzumab Plus Taxane for Human Epidermal Growth Factor Receptor 2-Positive, Advanced Breast Cancer: Primary Results From the Phase III MARIANNE Study. Pubmed ID 28056202. J Clin Oncol. 2017 Jan 10;35(2):141-148. doi: 10.1200/JCO.2016.67.4887. |
| Cloughesy T, Finocchiaro G, Belda-Iniesta C, Recht L, Brandes AA, Pineda E, Mikkelsen T, Chinot OL, Balana C, Macdonald DR, Westphal M, Hopkins K, Weller M, Bais C, Sandmann T, Bruey JM, Koeppen H, Liu B, Verret W, Phan SC, Shames DS. Randomized, Double-Blind, Placebo-Controlled, Multicenter Phase II Study of Onartuzumab Plus Bevacizumab Versus Placebo Plus Bevacizumab in Patients With Recurrent Glioblastoma: Efficacy, Safety, and Hepatocyte Growth Factor and O(6)-Methylguanine-DNA Methyltransferase Biomarker Analyses. Pubmed ID 27918718. J Clin Oncol. 2017 Jan 20;35(3):343-351. doi: 10.1200/JCO.2015.64.7685. |
| Spigel DR, Edelman MJ, O'Byrne K, Paz-Ares L, Mocci S, Phan S, Shames DS, Smith D, Yu W, Paton VE, Mok T. Results From the Phase III Randomized Trial of Onartuzumab Plus Erlotinib Versus Erlotinib in Previously Treated Stage IIIB or IV Non-Small-Cell Lung Cancer: METLung. Pubmed ID 27937096. J Clin Oncol. 2017 Feb;35(4):412-420. doi: 10.1200/JCO.2016.69.2160. |
| Pigneux A, Béné MC, Guardiola P, Recher C, Hamel JF, Sauvezie M, Harousseau JL, Tournilhac O, Witz F, Berthou C, Escoffre-Barbe M, Guyotat D, Fegueux N, Himberlin C, Hunault M, Delain M, Lioure B, Jourdan E, Bauduer F, Dreyfus F, Cahn JY, Sotto JJ, et al. Addition of Androgens Improves Survival in Elderly Patients With Acute Myeloid Leukemia: A GOELAMS Study. Pubmed ID 28129526. J Clin Oncol. 2017 Feb;35(4):387-393. doi: 10.1200/JCO.2016.67.6213. |
| van Imhoff GW, McMillan A, Matasar MJ, Radford J, Ardeshna KM, Kuliczkowski K, Kim W, Hong X, Goerloev JS, Davies A, Barrigón MDC, Ogura M, Leppä S, Fennessy M, Liao Q, van der Holt B, Lisby S, Hagenbeek A. Ofatumumab Versus Rituximab Salvage Chemoimmunotherapy in Relapsed or Refractory Diffuse Large B-Cell Lymphoma: The ORCHARRD Study. Pubmed ID 28029326. J Clin Oncol. 2017 Feb 10;35(5):544-551. doi: 10.1200/JCO.2016.69.0198. |
| Platzbecker U, Avvisati G, Cicconi L, Thiede C, Paoloni F, Vignetti M, Ferrara F, Divona M, Albano F, Efficace F, Fazi P, Sborgia M, Di Bona E, Breccia M, Borlenghi E, Cairoli R, Rambaldi A, Melillo L, La Nasa G, Fiedler W, Brossart P, Hertenstein B, et al. Improved Outcomes With Retinoic Acid and Arsenic Trioxide Compared With Retinoic Acid and Chemotherapy in Non-High-Risk Acute Promyelocytic Leukemia: Final Results of the Randomized Italian-German APL0406 Trial. Pubmed ID 27400939. J Clin Oncol. 2017 Feb 20;35(6):605-612. doi: 10.1200/JCO.2016.67.1982. |
| Choueiri TK, Halabi S, Sanford BL, Hahn O, Michaelson MD, Walsh MK, Feldman DR, Olencki T, Picus J, Small EJ, Dakhil S, George DJ, Morris MJ. Cabozantinib Versus Sunitinib As Initial Targeted Therapy for Patients With Metastatic Renal Cell Carcinoma of Poor or Intermediate Risk: The Alliance A031203 CABOSUN Trial. Pubmed ID 28199818. J Clin Oncol. 2017 Feb 20;35(6):591-597. doi: 10.1200/JCO.2016.70.7398. |
| Agarwala SS, Lee SJ, Yip W, Rao UN, Tarhini AA, Cohen GI, Reintgen DS, Evans TL, Brell JM, Albertini MR, Atkins MB, Dakhil SR, Conry RM, Sosman JA, Flaherty LE, Sondak VK, Carson WE, Smylie MG, Pappo AS, Kefford RF, Kirkwood JM. Phase III Randomized Study of 4 Weeks of High-Dose Interferon-Î±-2b in Stage T2bNO, T3a-bNO, T4a-bNO, and T1-4N1a-2a (microscopic) Melanoma: A Trial of the Eastern Cooperative Oncology Group-American College of Radiology Imaging Network Cancer Research Group (E1697). Pubmed ID 28135150. J Clin Oncol. 2017 Mar 10;35(8):885-892. doi: 10.1200/JCO.2016.70.2951. |
| Smith I, Yardley D, Burris H, De Boer R, Amadori D, McIntyre K, Ejlertsen B, Gnant M, Jonat W, Pritchard KI, Dowsett M, Hart L, Poggio S, Comarella L, Salomon H, Wamil B, O'Shaughnessy J. Comparative Efficacy and Safety of Adjuvant Letrozole Versus Anastrozole in Postmenopausal Patients With Hormone Receptor-Positive, Node-Positive Early Breast Cancer: Final Results of the Randomized Phase III Femara Versus Anastrozole Clinical Evaluation (FACE) Trial. Pubmed ID 28113032. J Clin Oncol. 2017 Apr 1;35(10):1041-1048. doi: 10.1200/JCO.2016.69.2871. |
| Thomas X, de Botton S, Chevret S, Caillot D, Raffoux E, Lemasle E, Marolleau JP, Berthon C, Pigneux A, Vey N, Reman O, Simon M, Recher C, Cahn JY, Hermine O, Castaigne S, Celli-Lebras K, Ifrah N, Preudhomme C, Terré C, Dombret H. Randomized Phase II Study of Clofarabine-Based Consolidation for Younger Adults With Acute Myeloid Leukemia in First Remission. Pubmed ID 28221862. J Clin Oncol. 2017 Apr 10;35(11):1223-1230. doi: 10.1200/JCO.2016.70.4551. |
| Scott BL, Pasquini MC, Logan BR, Wu J, Devine SM, Porter DL, Maziarz RT, Warlick ED, Fernandez HF, Alyea EP, Hamadani M, Bashey A, Giralt S, Geller NL, Leifer E, Le-Rademacher J, Mendizabal AM, Horowitz MM, Deeg HJ, Horwitz ME. Myeloablative Versus Reduced-Intensity Hematopoietic Cell Transplantation for Acute Myeloid Leukemia and Myelodysplastic Syndromes. Pubmed ID 28380315. J Clin Oncol. 2017 Apr 10;35(11):1154-1161. doi: 10.1200/JCO.2016.70.7091. |
| Tiseo M, Boni L, Ambrosio F, Camerini A, Baldini E, Cinieri S, Brighenti M, Zanelli F, Defraia E, Chiari R, Dazzi C, Tibaldi C, Turolla GM, D'Alessandro V, Zilembo N, Trolese AR, Grossi F, Riccardi F, Ardizzoni A. Italian, Multicenter, Phase III, Randomized Study of Cisplatin Plus Etoposide With or Without Bevacizumab as First-Line Treatment in Extensive-Disease Small-Cell Lung Cancer: The GOIRC-AIFA FARM6PMFJM Trial. Pubmed ID 28135143. J Clin Oncol. 2017 Apr 20;35(12):1281-1287. doi: 10.1200/JCO.2016.69.4844. |
| Seckl MJ, Ottensmeier CH, Cullen M, Schmid P, Ngai Y, Muthukumar D, Thompson J, Harden S, Middleton G, Fife KM, Crosse B, Taylor P, Nash S, Hackshaw A. Multicenter, Phase III, Randomized, Double-Blind, Placebo-Controlled Trial of Pravastatin Added to First-Line Standard Chemotherapy in Small-Cell Lung Cancer (LUNGSTAR). Pubmed ID 28240967. J Clin Oncol. 2017 May 10;35(14):1506-1514. doi: 10.1200/JCO.2016.69.7391. |
| Mason MD, Clarke NW, James ND, Dearnaley DP, Spears MR, Ritchie AWS, Attard G, Cross W, Jones RJ, Parker CC, Russell JM, Thalmann GN, Schiavone F, Cassoly E, Matheson D, Millman R, Rentsch CA, Barber J, Gilson C, Ibrahim A, Logue J, Lydon A, et al. Adding Celecoxib With or Without Zoledronic Acid for Hormone-Naïve Prostate Cancer: Long-Term Survival Results From an Adaptive, Multiarm, Multistage, Platform, Randomized Controlled Trial. Pubmed ID 28300506. J Clin Oncol. 2017 May 10;35(14):1530-1541. doi: 10.1200/JCO.2016.69.0677. |
| Bradstock KF, Link E, Di Iulio J, Szer J, Marlton P, Wei AH, Enno A, Schwarer A, Lewis ID, D'Rozario J, Coyle L, Cull G, Campbell P, Leahy MF, Hahn U, Cannell P, Tiley C, Lowenthal RM, Moore J, Cartwright K, Cunningham I, Taper J, et al. Idarubicin Dose Escalation During Consolidation Therapy for Adult Acute Myeloid Leukemia. Pubmed ID 28368672. J Clin Oncol. 2017 May 20;35(15):1678-1685. doi: 10.1200/JCO.2016.70.6374. |
| Yao JC, Guthrie KA, Moran C, Strosberg JR, Kulke MH, Chan JA, LoConte N, McWilliams RR, Wolin EM, Mattar B, McDonough S, Chen H, Blanke CD, Hochster HS. Phase III Prospective Randomized Comparison Trial of Depot Octreotide Plus Interferon Alfa-2b Versus Depot Octreotide Plus Bevacizumab in Patients With Advanced Carcinoid Tumors: SWOG S0518. Pubmed ID 28384065. J Clin Oncol. 2017 May 20;35(15):1695-1703. doi: 10.1200/JCO.2016.70.4072. |
| Jones RJ, Hussain SA, Protheroe AS, Birtle A, Chakraborti P, Huddart RA, Jagdev S, Bahl A, Stockdale A, Sundar S, Crabb SJ, Dixon-Hughes J, Alexander L, Morris A, Kelly C, Stobo J, Paul J, Powles T. Randomized Phase II Study Investigating Pazopanib Versus Weekly Paclitaxel in Relapsed or Progressive Urothelial Cancer. Pubmed ID 28402747. J Clin Oncol. 2017 Jun 1;35(16):1770-1777. doi: 10.1200/JCO.2016.70.7828. |
| Catton CN, Lukka H, Gu CS, Martin JM, Supiot S, Chung PWM, Bauman GS, Bahary JP, Ahmed S, Cheung P, Tai KH, Wu JS, Parliament MB, Tsakiridis T, Corbett TB, Tang C, Dayes IS, Warde P, Craig TK, Julian JA, Levine MN. Randomized Trial of a Hypofractionated Radiation Regimen for the Treatment of Localized Prostate Cancer. Pubmed ID 28296582. J Clin Oncol. 2017 Jun 10;35(17):1884-1890. doi: 10.1200/JCO.2016.71.7397. |
| Zucca E, Conconi A, Martinelli G, Bouabdallah R, Tucci A, Vitolo U, Martelli M, Pettengell R, Salles G, Sebban C, Guillermo AL, Pinotti G, Devizzi L, Morschhauser F, Tilly H, Torri V, Hohaus S, Ferreri AJM, Zachée P, Bosly A, Haioun C, Stelitano C, et al. Final Results of the IELSG-19 Randomized Trial of Mucosa-Associated Lymphoid Tissue Lymphoma: Improved Event-Free and Progression-Free Survival With Rituximab Plus Chlorambucil Versus Either Chlorambucil or Rituximab Monotherapy. Pubmed ID 28355112. J Clin Oncol. 2017 Jun 10;35(17):1905-1912.doi:10.1200/JCO.2016.70.6994. |
| Arcangeli G, Saracino B, Arcangeli S, Gomellini S, Petrongari MG, Sanguineti G, Strigari L. Moderate Hypofractionation in High-Risk, Organ-Confined Prostate Cancer: Final Results of a Phase III Randomized Trial. Pubmed ID 28355113. J Clin Oncol. 2017 Jun 10;35(17):1891-1897. doi: 10.1200/JCO.2016.70.4189. |

| **The Lancet** |
| --- |
| Bruix J, Qin S, Merle P, Granito A, Huang YH, Bodoky G, Pracht M, Yokosuka O, Rosmorduc O, Breder V, Gerolami R, Masi G, Ross PJ, Song T, Bronowicki JP, Ollivier-Hourmand I, Kudo M, Cheng AL, Llovet JM, Finn RS, LeBerre MA, Baumhauer A, et al. Regorafenib for patients with hepatocellular carcinoma who progressed on sorafenib treatment (RESORCE): a randomised, double-blind, placebo-controlled, phase 3 trial. Pubmed ID 27932229. Lancet. 2017 Jan 7;389(10064):56-66. doi: 10.1016/S0140-6736(16)32453-9. |
| Rittmeyer A, Barlesi F, Waterkamp D, Park K, Ciardiello F, von Pawel J, Gadgeel SM, Hida T, Kowalski DM, Dols MC, Cortinovis DL, Leach J, Polikoff J, Barrios C, Kabbinavar F, Frontera OA, De Marinis F, Turna H, Lee JS, Ballinger M, Kowanetz M, He P, et al. Atezolizumab versus docetaxel in patients with previously treated non-small-cell lung cancer (OAK): a phase 3, open-label, multicentre randomised controlled trial. Pubmed ID 27979383. Lancet. 2017 Jan 21;389(10066):255-265. doi: 10.1016/S0140-6736(16)32517-X. |
| Durie BG, Hoering A, Abidi MH, Rajkumar SV, Epstein J, Kahanic SP, Thakuri M, Reu F, Reynolds CM, Sexton R, Orlowski RZ, Barlogie B, Dispenzieri A. Bortezomib with lenalidomide and dexamethasone versus lenalidomide and dexamethasone alone in patients with newly diagnosed myeloma without intent for immediate autologous stem-cell transplant (SWOG S0777): a randomised, open-label, phase 3 trial. Pubmed ID 28017406. Lancet. 2017 Feb 4;389(10068):519-527. doi: 10.1016/S0140-6736(16)31594-X. |
| Soria JC, Tan DSW, Chiari R, Wu YL, Paz-Ares L, Wolf J, Geater SL, Orlov S, Cortinovis D, Yu CJ, Hochmair M, Cortot AB, Tsai CM, Moro-Sibilot D, Campelo RG, McCulloch T, Sen P, Dugan M, Pantano S, Branle F, Massacesi C, de Castro G Jr. First-line ceritinib versus platinum-based chemotherapy in advanced ALK-rearranged non-small-cell lung cancer (ASCEND-4): a randomised, open-label, phase 3 study. Pubmed ID 28126333. Lancet. 2017 Mar 4;389(10072):917-929. doi: 10.1016/S0140-6736(17)30123-X. |
| Kepreotes E, Whitehead B, Attia J, Oldmeadow C, Collison A, Searles A, Goddard B, Hilton J, Lee M, Mattes J. High-flow warm humidified oxygen versus standard low-flow nasal cannula oxygen for moderate bronchiolitis (HFWHO RCT): an open, phase 4, randomised controlled trial. Pubmed ID 28161016. Lancet. 2017 Mar 4;389(10072):930-939. doi: 10.1016/S0140-6736(17)30061-2. |
| Neoptolemos JP, Palmer DH, Ghaneh P, Psarelli EE, Valle JW, Halloran CM, Faluyi O, O'Reilly DA, Cunningham D, Wadsley J, Darby S, Meyer T, Gillmore R, Anthoney A, Lind P, Glimelius B, Falk S, Izbicki JR, Middleton GW, Cummins S, Ross PJ, Wasan H, et al. Comparison of adjuvant gemcitabine and capecitabine with gemcitabine monotherapy in patients with resected pancreatic cancer (ESPAC-4): a multicentre, open-label, randomised, phase 3 trial. Pubmed ID 28129987. Lancet. 2017 Mar 11;389(10073):1011-1024. doi: 10.1016/S0140-6736(16)32409-6. |
| Cameron D, Piccart-Gebhart MJ, Gelber RD, Procter M, Goldhirsch A, de Azambuja E, Castro G Jr, Untch M, Smith I, Gianni L, Baselga J, Al-Sakaff N, Lauer S, McFadden E, Leyland-Jones B, Bell R, Dowsett M, Jackisch C; Herceptin Adjuvant (HERA) Trial Study Team. 11 years' follow-up of trastuzumab after adjuvant chemotherapy in HER2-positive early breast cancer: final analysis of the HERceptin Adjuvant (HERA) trial. Pubmed ID 28215665. Lancet. 2017 Mar 25;389 (10075):1195-1205. doi: 10.1016/S0140-6736(16)32616-2. |
| Atkin W, Wooldrage K, Parkin DM, Kralj-Hans I, MacRae E, Shah U, Duffy S, Cross AJ. Long term effects of once-only flexible sigmoidoscopy screening after 17 years of follow-up: the UK Flexible Sigmoidoscopy Screening randomised controlled trial. Pubmed ID 28236467. Lancet. 2017 Apr 1;389(10076):1299-1311. doi: 10.1016/S0140-6736(17)30396-3. |
| le Roux CW, Astrup A, Fujioka K, Greenway F, Lau DCW, Van Gaal L, Ortiz RV, Wilding JPH, Skjøth TV, Manning LS, Pi-Sunyer X; SCALE Obesity Prediabetes NN8022-1839 Study Group. 3 years of liraglutide versus placebo for type 2 diabetes risk reduction and weight management in individuals with prediabetes: a randomised, double-blind trial. Pubmed ID 28237263. Lancet. 2017 Apr 8;389(10077):1399-1409. doi: 10.1016/S0140-6736(17)30069-7. |
| Fixation using Alternative Implants for the Treatment of Hip fractures (FAITH) Investigators. Fracture fixation in the operative management of hip fractures (FAITH): an international, multicentre, randomised controlled trial. Pubmed ID 28262269. Lancet. 2017 Apr 15;389(10078):1519-1527. doi: 10.1016/S0140-6736(17)30066-1. |
| Ohman EM, Roe MT, Steg PG, James SK, Povsic TJ, White J, Rockhold F, Plotnikov A, Mundl H, Strony J, Sun X, Husted S, Tendera M, Montalescot G, Bahit MC, Ardissino D, Bueno H, Claeys MJ, Nicolau JC, Cornel JH, Goto S, Kiss RG, et al. Clinically significant bleeding with low-dose rivaroxaban versus aspirin, in addition to P2Y12 inhibition, in acute coronary syndromes (GEMINI-ACS-1): a double-blind, multicentre, randomised trial. Pubmed ID 28325638. Lancet. 2017 May 6;389(10081):1799-1808. doi: 10.1016/S0140-6736(17)30751-1. |
| Chan FKL, Ching JYL, Tse YK, Lam K, Wong GLH, Ng SC, Lee V, Au KWL, Cheong PK, Suen BY, Chan H, Kee KM, Lo A, Wong VWS, Wu JCY, Kyaw MH. Gastrointestinal safety of celecoxib versus naproxen in patients with cardiothrombotic diseases and arthritis after upper gastrointestinal bleeding (CONCERN): an industry-independent, double-blind, double-dummy, randomised trial. Pubmed ID 28410791. Lancet. 2017 Jun 17;389(10087):2375-2382. doi: 10.1016/S0140-6736(17)30981-9. |

| **New England Journal of Medicine** |
| --- |
| Hiatt WR, Fowkes FG, Heizer G, Berger JS, Baumgartner I, Held P, Katona BG, Mahaffey KW, Norgren L, Jones WS, Blomster J, Millegård M, Reist C, Patel MR; EUCLID Trial Steering Committee and Investigators. Ticagrelor versus Clopidogrel in Symptomatic Peripheral Artery Disease. Pubmed ID 27959717. N Engl J Med. 2017 Jan 5;376(1):32-40. doi: 10.1056/NEJMoa1611688. |
| Strosberg J, El-Haddad G, Wolin E, Hendifar A, Yao J, Chasen B, Mittra E, Kunz PL, Kulke MH, Jacene H, Bushnell D, O'Dorisio TM, Baum RP, Kulkarni HR, Caplin M, Lebtahi R, Hobday T, Delpassand E, Van Cutsem E, Benson A, Srirajaskanthan R, Pavel M, et al. Phase 3 Trial of (177)Lu-Dotatate for Midgut Neuroendocrine Tumors. Pubmed ID 28076709. N Engl J Med. 2017 Jan 12;376(2):125-135. doi: 10.1056/NEJMoa1607427. |
| Montalban X, Hauser SL, Kappos L, Arnold DL, Bar-Or A, Comi G, de Seze J, Giovannoni G, Hartung HP, Hemmer B, Lublin F, Rammohan KW, Selmaj K, Traboulsee A, Sauter A, Masterman D, Fontoura P, Belachew S, Garren H, Mairon N, Chin P, Wolinsky JS, et al. Ocrelizumab versus Placebo in Primary Progressive Multiple Sclerosis. Pubmed ID 28002688. N Engl J Med. 2017 Jan 19;376(3):209-220. doi: 10.1056/NEJMoa1606468. |
| Mehra MR, Naka Y, Uriel N, Goldstein DJ, Cleveland JC Jr, Colombo PC, Walsh MN, Milano CA, Patel CB, Jorde UP, Pagani FD, Aaronson KD, Dean DA, McCants K, Itoh A, Ewald GA, Horstmanshof D, Long JW, Salerno C; MOMENTUM 3 Investigators. A Fully Magnetically Levitated Circulatory Pump for Advanced Heart Failure. Pubmed ID 27959709. N Engl J Med. 2017 Feb 2;376(5):440-450. doi: 10.1056/NEJMoa1610426. |
| Rogers JG, Pagani FD, Tatooles AJ, Bhat G, Slaughter MS, Birks EJ, Boyce SW, Najjar SS, Jeevanandam V, Anderson AS, Gregoric ID, Mallidi H, Leadley K, Aaronson KD, Frazier OH, Milano CA. Intrapericardial Left Ventricular Assist Device for Advanced Heart Failure. Pubmed ID 28146651. N Engl J Med. 2017 Feb 2;376(5):451-460. doi: 10.1056/NEJMoa1602954. |
| Shipley WU, Seiferheld W, Lukka HR, Major PP, Heney NM, Grignon DJ, Sartor O, Patel MP, Bahary JP, Zietman AL, Pisansky TM, Zeitzer KL, Lawton CA, Feng FY, Lovett RD, Balogh AG, Souhami L, Rosenthal SA, Kerlin KJ, Dignam JJ, Pugh SL, Sandler HM, et al. Radiation with or without Antiandrogen Therapy in Recurrent Prostate Cancer. Pubmed ID 28146658. N Engl J Med. 2017 Feb 2;376(5):417-428. doi: 10.1056/NEJMoa1607529. |
| Mok TS, Wu Y-L, Ahn M-J, Garassino MC, Kim HR, Ramalingam SS, Shepherd FA, He Y, Akamatsu H, Theelen WS, Lee CK, Sebastian M, Templeton A, Mann H, Marotti M, Ghiorghiu S, Papadimitrakopoulou VA; AURA3 Investigators. Osimertinib or Platinum-Pemetrexed in EGFR T790M-Positive Lung Cancer. Pubmed ID 27959700. N Engl J Med. 2017 Feb 16;376(7):629-640. doi: 10.1056/NEJMoa1612674. |
| Agus MS, Wypij D, Hirshberg EL, Srinivasan V, Faustino EV, Luckett PM, Alexander JL, Asaro LA, Curley MA, Steil GM, Nadkarni VM; HALF-PINT Study Investigators and the PALISI Network. Tight Glycemic Control in Critically Ill Children. Pubmed ID 28118549. N Engl J Med. 2017 Feb 23;376(8):729-741. doi: 10.1056/NEJMoa1612348. |
| Kantarjian H, Stein A, Gökbuget N, Fielding AK, Schuh AC, Ribera JM, Wei A, Dombret H, Foà  R, Bassan R, Arslan Önder, Sanz MA, Bergeron J, Demirkan F, Lech-Maranda E, Rambaldi A, Thomas X, Horst HA, Brüggemann M, Klapper W, Wood BL, Fleishman A, et al. Blinatumomab versus Chemotherapy for Advanced Acute Lymphoblastic Leukemia. Pubmed ID 28249141. N Engl J Med. 2017 Mar 2;376(9):836-847. doi: 10.1056/NEJMoa1609783. |
| Bellmunt J, de Wit R, Vaughn DJ, Fradet Y, Lee JL, Fong L, Vogelzang NJ, Climent MA, Petrylak DP, Choueiri TK, Necchi A, Gerritsen W, Gurney H, Quinn DI, Culine S, Sternberg CN, Mai Y, Poehlein CH, Perini RF, Bajorin DF; KEYNOTE-045 Investigators. Pembrolizumab as Second-Line Therapy for Advanced Urothelial Carcinoma. Pubmed ID 28212060. N Engl J Med. 2017 Mar 16;376(11):1015-1026. doi: 10.1056/NEJMoa1613683. |
| Perry JR, Laperriere N, O'Callaghan CJ, Brandes AA, Menten J, Phillips C, Fay M, Nishikawa R, Cairncross JG, Roa W, Osoba D, Rossiter JP, Sahgal A, Hirte H, Laigle-Donadey F, Franceschi E, Chinot O, Golfinopoulos V, Fariselli L, Wick A, Feuvret L, Back M, et al. Short-Course Radiation plus Temozolomide in Elderly Patients with Glioblastoma. Pubmed ID 28296618. N Engl J Med. 2017 Mar 16;376(11):1027-1037. doi: 10.1056/NEJMoa1611977. |
| Weitz JI, Lensing AWA, Prins MH, Bauersachs R, Beyer-Westendorf J, Bounameaux H, Brighton TA, Cohen AT, Davidson BL, Decousus H, Freitas MCS, Holberg G, Kakkar AK, Haskell L, van Bellen B, Pap AF, Berkowitz SD, Verhamme P, Wells PS, Prandoni P; EINSTEIN CHOICE Investigators. Rivaroxaban or Aspirin for Extended Treatment of Venous Thromboembolism. Pubmed ID 28316279. N Engl J Med. 2017 Mar 30;376(13):1211-1222. doi: 10.1056/NEJMoa1700518. |
| Smits PC, Abdel-Wahab M, Neumann FJ, Boxma-de Klerk BM, Lunde K, Schotborgh CE, Piroth Z, Horak D, Wlodarczak A, Ong PJ, Hambrecht R, Angerås O, Richardt G, Omerovic E; Compare-Acute Investigators. Fractional Flow Reserve-Guided Multivessel Angioplasty in Myocardial Infarction. Pubmed ID 28317428. N Engl J Med. 2017 Mar 30;376(13):1234-1244. doi: 10.1056/NEJMoa1701067. |
| Attal M, Lauwers-Cances V, Hulin C, Leleu X, Caillot D, Escoffre M, Arnulf B, Macro M, Belhadj K, Garderet L, Roussel M, Payen C, Mathiot C, Fermand JP, Meuleman N, Rollet S, Maglio ME, Zeytoonjian AA, Weller EA, Munshi N, Anderson KC, Richardson PG, et al. Lenalidomide, Bortezomib, and Dexamethasone with Transplantation for Myeloma. Pubmed ID 28379796. N Engl J Med. 2017 Apr 6;376(14):1311-1320. doi: 10.1056/NEJMoa1611750. |
| Ridker PM, Revkin J, Amarenco P, Brunell R, Curto M, Civeira F, Flather M, Glynn RJ, Gregoire J, Jukema JW, Karpov Y, Kastelein JJP, Koenig W, Lorenzatti A, Manga P, Masiukiewicz U, Miller M, Mosterd A, Murin J, Nicolau JC, Nissen S, Ponikowski P, et al. Cardiovascular Efficacy and Safety of Bococizumab in High-Risk Patients. Pubmed ID 28304242. N Engl J Med. 2017 Apr 20;376(16):1527-1539. doi: 10.1056/NEJMoa1701488. |
| Ramanan AV, Dick AD, Jones AP, McKay A, Williamson PR, Compeyrot-Lacassagne S, Hardwick B, Hickey H, Hughes D, Woo P, Benton D, Edelsten C, Beresford MW; SYCAMORE Study Group. Adalimumab plus Methotrexate for Uveitis in Juvenile Idiopathic Arthritis. Pubmed ID 28445659. N Engl J Med. 2017 Apr 27;376(17):1637-1646. doi: 10.1056/NEJMoa1614160. |
| Sabatine MS, Giugliano RP, Keech AC, Honarpour N, Wiviott SD, Murphy SA, Kuder JF, Wang H, Liu T, Wasserman SM, Sever PS, Pedersen TR; FOURIER Steering Committee and Investigators. Evolocumab and Clinical Outcomes in Patients with Cardiovascular Disease. Pubmed ID 28304224. N Engl J Med. 2017 May 4;376(18):1713-1722. doi: 10.1056/NEJMoa1615664. |
| Packer M, O'Connor C, McMurray JJV, Wittes J, Abraham WT, Anker SD, Dickstein K, Filippatos G, Holcomb R, Krum H, Maggioni AP, Mebazaa A, Peacock WF, Petrie MC, Ponikowski P, Ruschitzka F, van Veldhuisen DJ, Kowarski LS, Schactman M, Holzmeister J; TRUE-AHF Investigators. Effect of Ularitide on Cardiovascular Mortality in Acute Heart Failure. Pubmed ID 28402745. N Engl J Med. 2017 May 18;376(20):1956-1964. doi: 10.1056/NEJMoa1601895. |
| Lincoff AM, Nicholls SJ, Riesmeyer JS, Barter PJ, Brewer HB, Fox KAA, Gibson CM, Granger C, Menon V, Montalescot G, Rader D, Tall AR, McErlean E, Wolski K, Ruotolo G, Vangerow B, Weerakkody G, Goodman SG, Conde D, McGuire DK, Nicolau JC, Leiva-Pons JL, et al. Evacetrapib and Cardiovascular Outcomes in High-Risk Vascular Disease. Pubmed ID 28514624. N Engl J Med. 2017 May 18;376(20):1933-1942. doi: 10.1056/NEJMoa1609581. |
| Masuda N, Lee SJ, Ohtani S, Im YH, Lee ES, Yokota I, Kuroi K, Im SA, Park BW, Kim SB, Yanagita Y, Ohno S, Takao S, Aogi K, Iwata H, Jeong J, Kim A, Park KH, Sasano H, Ohashi Y, Toi M. Adjuvant Capecitabine for Breast Cancer after Preoperative Chemotherapy. Pubmed ID 28564564. N Engl J Med. 2017 Jun 1;376(22):2147-2159. doi: 10.1056/NEJMoa1612645. |
| Faries MB, Thompson JF, Cochran AJ, Andtbacka RH, Mozzillo N, Zager JS, Jahkola T, Bowles TL, Testori A, Beitsch PD, Hoekstra HJ, Moncrieff M, Ingvar C, Wouters MWJM, Sabel MS, Levine EA, Agnese D, Henderson M, Dummer R, Rossi CR, Neves RI, Trocha SD, et al. Completion Dissection or Observation for Sentinel-Node Metastasis in Melanoma. Pubmed ID 28591523. N Engl J Med. 2017 Jun 8;376(23):2211-2222. doi: 10.1056/NEJMoa1613210. |
| Wykrzykowska JJ, Kraak RP, Hofma SH, van der Schaaf RJ, Arkenbout EK, IJsselmuiden AJ, Elias J, van Dongen IM, Tijssen RYG, Koch KT, Baan J Jr, Vis MM, de Winter RJ, Piek JJ, Tijssen JGP, Henriques JPS; AIDA Investigators. Bioresorbable Scaffolds versus Metallic Stents in Routine PCI Pubmed ID 28402237. N Engl J Med. 2017 Jun 15;376(24):2319-2328. doi: 10.1056/ NEJMoa1614954. |
| Kraft WK, Adeniyi-Jones SC, Chervoneva I, Greenspan JS, Abatemarco D, Kaltenbach K, Ehrlich ME. Buprenorphine for the Treatment of the Neonatal Abstinence Syndrome Pubmed ID 28468518. N Engl J Med. 2017 Jun 15;376(24):2341-2348. doi: 10.1056/NEJMoa1614835. |
| Carbone DP, Reck M, Paz-Ares L, Creelan B, Horn L, Steins M, Felip E, van den Heuvel MM, Ciuleanu TE, Badin F, Ready N, Hiltermann TJN, Nair S, Juergens R, Peters S, Minenza E, Wrangle JM, Rodriguez-Abreu D, Borghaei H, Blumenschein GR Jr, Villaruz LC, Havel L, et al. First-Line Nivolumab in Stage IV or Recurrent Non-Small-Cell Lung Cancer. Pubmed ID 28636851. N Engl J Med. 2017 Jun 22;376(25):2415-2426. doi: 10.1056/NEJMoa1613493. |
| von Minckwitz G, Procter M, de Azambuja E, Zardavas D, Benyunes M, Viale G, Suter T, Arahmani A, Rouchet N, Clark E, Knott A, Lang I, Levy C, Yardley DA, Bines J, Gelber RD, Piccart M, Baselga J; APHINITY Steering Committee and Investigators. Adjuvant Pertuzumab and Trastuzumab in Early HER2-Positive Breast Cancer. Pubmed ID 28581356. N Engl J Med. 2017 Jul 13;377(2)122-131. doi: 10.1056/NEJMoa1703643. |
| Fizazi K, Tran N, Fein L, Matsubara N, Rodriguez-Antolin A, Alekseev BY, Özgûroğlu M, Ye D, Feyerabend S, Protheroe A, De Porre P, Kheoh T, Park YC, Todd MB, Chi KN; LATITUDE Investigators. Abiraterone plus Prednisone in Metastatic, Castration-Sensitive Prostate Cancer. Pubmed ID 28578607. N Engl J Med. 2017 Jul 27;377(4):352-360. doi: 10.1056/NEJMoa1704174. |
| James ND, de Bono JS, Spears MR, Clarke NW, Mason MD, Dearnaley DP, Ritchie AWS, Amos CL, Gilson C, Jones RJ, Matheson D, Millman R, Attard G, Chowdhury S, Cross WR, Gillessen S, Parker CC, Russell JM, Berthold DR, Brawley C, Adab F, Aung S, et al. Abiraterone for Prostate Cancer Not Previously Treated with Hormone Therapy. Pubmed ID 28578639. N Engl J Med. 2017 Jul 27;377(4):338-351. doi: 10.1056/NEJMoa1702900. |
| Stone RM, Mandrekar SJ, Sanford BL, Laumann K, Geyer S, Bloomfield CD, Thiede C, Prior TW, Döhner K, Marcucci G, Lo-Coco F, Klisovic RB, Wei A, Sierra J, Sanz MA, Brandwein JM, de Witte T, Niederwieser D, Appelbaum FR, Medeiros BC, Tallman MS, Krauter J, et al. Midostaurin plus Chemotherapy for Acute Myeloid Leukemia with a FLT3 Mutation. Pubmed ID 28644114. N Engl J Med. 2017 Aug 3;377(5):454-464. doi: 10.1056/NEJMoa1614359. |
| Robson M, Im SA, Senkus E, Xu B, Domchek SM, Masuda N, Delaloge S, Li W, Tung N, Armstrong A, Wu W, Goessl C, Runswick S, Conte P. Olaparib for Metastatic Breast Cancer in Patients with a Germline BRCA Mutation. Pubmed ID 28578601. N Engl J Med. 2017 Aug 10;377(6):523-533. doi: 10.1056/NEJMoa1706450. |
| Neal B, Perkovic V, Mahaffey KW, de Zeeuw D, Fulcher G, Erondu N, Shaw W, Law G, Desai M, Matthews DR; CANVAS Program Collaborative Group. Canagliflozin and Cardiovascular and Renal Events in Type 2 Diabetes. Pubmed ID 28605608. N Engl J Med. 2017 Aug 17;377(7):644-657. doi: 10.1056/NEJMoa1611925. |
| Marso SP, McGuire DK, Zinman B, Poulter NR, Emerson SS, Pieber TR, Pratley RE, Haahr PM, Lange M, Brown-Frandsen K, Moses A, Skibsted S, Kvist K, Buse JB; DEVOTE Study Group.. Efficacy and Safety of Degludec versus Glargine in Type 2 Diabetes. Pubmed ID 28605603. N Engl J Med. 2017 Aug 24;377(8):723-732. doi: 10.1056/NEJMoa1615692. |
| Peters S, Camidge DR, Shaw AT, Gadgeel S, Ahn JS, Kim DW, Ou SI, Pérol M, Dziadziuszko R, Rosell R, Zeaiter A, Mitry E, Golding S, Balas B, Noe J, Morcos PN, Mok T; ALEX Trial Investigators. Alectinib versus Crizotinib in Untreated ALK-Positive Non-Small-Cell Lung Cancer. Pubmed ID 28586279. N Engl J Med. 2017 Aug 31;377(9):829-838. doi: 10.1056/NEJMoa1704795. |
